# The Obstetrician’s Toilet: Hints by Professor Simpson

**Published:** 1851-10

**Authors:** 


*The Obstetrician's Toilet: Hints by* PROFESSOR SIMPSON.—In a recent discussion upon Puerperal Fever, before the Medico-Chirurgical Society of Edinburgh, Dr. Simpson urged "the great importance of carefully ridding the fingers from all matters in the least degree likely to prove hurtful, if inoculated into the vagina of a puerperal patient." Chloride of lime, he said, "has been recommended by various practitioners as a good wash for ridding the fingers from morbific matter." Dr. Simpson "has used for the same purpose for years, daily, (or rather, generally, often during the day,) a solution of cyanide of potassium, which is more effective even than chloride of lime; and it had this other advantage, that it removes, readily and at once, all such stains as the fingers of the accoucheur are apt to receive in treating uterine diseases—with nitrate of silver, iodine and the like.

Dr. Simpson objects to the use of gloves. "He had been informed of an instance by Professor Patterson, in which a medical gentleman, after having lost several cases of puerperal fever, got rid of the disease in his practice by changing his clothes, and using chloride of lime, etc.; but it again returned to him, when he happened to deliver a patient, immediately after wearing a pair of gloves which he had used during the time of the puerperal epidemic: and certainly, if there was any piece of dress more apt to retain the contagion than another, it was this useless and superfluous appendage to our attire."—*Edinbargh Monthly Journal,* July 1851.

